# Status, Alert System, and Prediction of Cyanobacterial Bloom in South Korea

**DOI:** 10.1155/2015/584696

**Published:** 2015-02-01

**Authors:** Ankita Srivastava, Chi-Yong Ahn, Ravi Kumar Asthana, Hyung-Gwan Lee, Hee-Mock Oh

**Affiliations:** ^1^Environmental Biotechnology Research Center, Korea Research Institute of Bioscience and Biotechnology, Daejeon 305-806, Republic of Korea; ^2^Centre of Advanced Study in Botany, Banaras Hindu University, Varanasi 221 005, India

## Abstract

Bloom-forming freshwater cyanobacterial genera pose a major ecological problem due to their ability to produce toxins and other bioactive compounds, which can have important implications in illnesses of humans and livestock. Cyanobacteria such as *Microcystis, Anabaena, Oscillatoria, Phormidium*, and *Aphanizomenon* species producing microcystins and anatoxin-a have been predominantly documented from most South Korean lakes and reservoirs. With the increase in frequency of such blooms, various monitoring approaches, treatment processes, and prediction models have been developed in due course. In this paper we review the field studies and current knowledge on toxin producing cyanobacterial species and ecological variables that regulate toxin production and bloom formation in major rivers (Han, Geum, Nakdong, and Yeongsan) and reservoirs in South Korea. In addition, development of new, fast, and high-throughput techniques for effective monitoring is also discussed with cyanobacterial bloom advisory practices, current management strategies, and their implications in South Korean freshwater bodies.

## 1. Introduction

**Figure 1:**
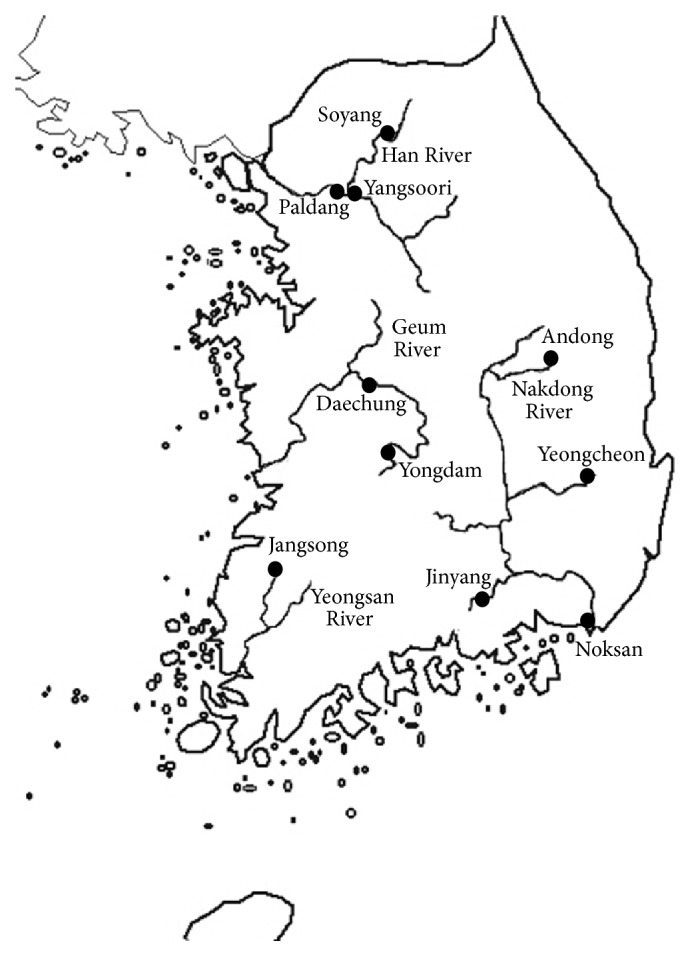
Map showing the location of four major rivers in South Korea.

Cyanobacteria can form dense blooms, scums, and mats that hamper the quality of water. Cyanobacterial genera like* Anabaena*,* Aphanizomenon*,* Cylindrospermopsis*,* Lyngbya*,* Microcystis*,* Oscillatoria*,* Nodularia,* and* Nostoc* are known to produce a wide variety of toxic compounds [1]. There have been increasing reports of cyanobacterial toxins and toxigenic species worldwide. Environmental factors that influence cyanobacteria dominance are well studied but the abundance of cyanobacteria varies with habitats as well as the environmental regimes. Favorable conditions for a cyanobacterial bloom include light, temperature, nutrients (mainly N and P), and quiescent water [2]. Eutrophication has been cited as a major cause of increasing cyanobacterial harmful algal blooms [3] and is also a widely recognized problem in South Korea [4, 5]. Four major rivers like Han, Geum, Nakdong, and Yeongsan in South Korea (Figure 1) are also suffering from cyanobacterial blooms. The Han River is the largest river system located in the central region and the Nakdong River is the second largest river system located in the southeastern part of the Korean peninsula. The Han River is the main water resource for the Seoul metropolitan area while the Nakdong River supplies water to Busan and Daegu, the second and third biggest cities, respectively. The water quality is deteriorated in the midstream and downstream regions where most of the population and major industries are located. The Geum River watershed and the Yeongsan River watershed are in the western part of the country. Most of the studies have focused on Daechung Reservoir which is located upstream of the Geum River and is the source of residential, agricultural, and industrial purposes.

In its Green Vision 21 river quality, 114 rivers and streams located in the four major basins of the Han, Nakdong, Geum, and Yeongsan were assigned Class I (i.e., best quality to 36 catchments) and Class II (46 catchments) quality. This grading system is based on variables such as biochemical oxygen demand (BOD), pH, suspended solids (SS), dissolved oxygen (DO), total phosphorus and nitrogen, and total* E.   coli* count.

Authorities responsible for water resources planning and management are often faced with problems of determining policy in accordance with a future climate change. Ministry of Environment started a national survey for streams and rivers health evaluation project in 2007 with a task of monitoring of stream sites for periphyton, benthic macroinvertebrates, fish, and riparian characteristics [6]. Recently, algal blooms caused a major scare over the quality of the country's drinking water. The Nakdong River and Han River were affected more seriously, which supply water to major cities in South Korea [7]. Long heat wave, drought, and plenty of sunlight with high levels of nutrients were reported as the main causes of blooms by the South Korean government.

Over the years, various field studies have been conducted for understanding the diverse interactions among physicochemical and biological variables leading to the proliferation of cyanobacterial blooms in Korean freshwater bodies [8, 9]. Furthermore, several monitoring approaches and predictive models were developed to provide accurate and timely information regarding the development of cyanobacterial bloom in the water bodies [10, 11]. Lee et al. [12] summarized various techniques that have been adopted for the control and mitigation of algal blooms in South Korea. This review presents the advances in the understanding of the occurrence and toxicity of cyanobacterial blooms in South Korean water bodies. Development of new, high-throughput techniques for effective monitoring, cyanobacterial bloom advisory practices, predictive models, and current management strategies are also discussed here.

## 2. Field Studies regarding Cyanobacterial Blooms in South Korea

South Korea relies on rivers and streams for water supply due to lack of reliable groundwater sources. Four major rivers (Han, Nakdong, Geum, and Yeongsan) meet the needs of more than 40 million residents. The induction of the growth and development of cyanobacterial blooms are regulated by various environmental factors [20]. Therefore, many researchers have focused on the detailed study of these factors and natural variability of toxins concentrations in various Korean reservoirs (Table 1).

Distribution of dominant species of cyanobacteria and the amount of hepatotoxic microcystins (MCs) and neurotoxic anatoxin-a in cyanobacterial blooms were investigated in 12 Korean lakes during 1992–1995 [13]. Six species each of* Microcystis* and* Anabaena* and two of* Oscillatoria* with percent dominance of 60, 30, and 10%, respectively, were identified in these lakes. MCs were identified as the main toxin and anatoxin-a was also reported for the first time from freshwater sources in South Korea. In another study, trophic status of ten reservoirs in the upstream and middle stream regions and three estuarine reservoirs was evaluated from 1993 to 1994 and from 1994 to 1995, respectively [21]. A seasonal variation in the pattern of phytoplankton standing crop was observed with higher density occurring in the estuarine reservoirs than deep upstream reservoirs due to high nutrient concentrations and seasonal changes in hydrology. Differences in the timing of succession were also reported in these reservoirs. Diatoms like* Asterionella* and* Aulacoseira* were dominant in spring (in deep reservoirs) and winter (in shallow reservoirs) while cyanobacteria (mostly* Microcystis*) appeared in estuarine reservoirs in warm seasons when there was a drought.

Various studies have focused on water quality, algal community composition, toxin production, short-term prediction of algal blooms, and variations in the environmental factors in the Daechung Reservoir [22–25]. In a study by Oh et al. [16], physico- and biochemical processes, along with changes in MC concentration, were monitored during the period of cyanobacterial blooms in Daechung Reservoir. Since MC analysis is important for determining the safety of water resources, an indirect monitoring method was proposed for estimating their concentrations in eutrophic waters based on phytoplankton number, chlorophyll-*a* (Chl* a*) concentration, and the ratio of the particulate and the dissolved forms of nitrogen (N) and phosphorus (P). The ratio of particulate to dissolved N or P at 0.6 could be used as a threshold for determining the MC concentration. The MC concentration also varied with the particulate N/P ratio. It was less than 50 ng/L at a particulate N/P ratio < 8, whereas it varied substantially at higher ratios. Relationship between anatoxin-a production and environmental factors was also analyzed in the reservoir [17]. Anatoxin-a was mainly produced by* Anabaena* sp. and* Oscillatoria* sp. and found to be highly correlated with N : P ratio. The magnitude and duration of rainfall also played an important role in determining the extent of cyanobacterial blooms in the Daechung Reservoir. The major species and relative abundance of cyanobacteria varied depending on the climatic conditions [26, 27]. The composition and dynamics of cyanobacteria during bloom were further elucidated using molecular-based techniques. Various genes, for example, 16S rRNA, internal transcribed spacer (ITS), and phycocyanin intergenic spacer (PC-IGS), were used for analyzing cyanobacterial diversity [28] and for characterizing toxic and nontoxic* Microcystis* colonies in natural populations [9, 29]. The ratio of toxigenic* Microcystis* sp. to that of total* Microcystis* sp. ranged from 7.6 to 56.6% and the proportion changes of potentially toxic* Microcystis* genotypes were more closely related with water temperature [9].

Ha et al. [30, 31] have shown the importance of flow regulation of dams on the proliferation and succession pattern of phytoplankton in the lower Nakdong River. Horizontal and vertical distributions of MCs were also examined for the first time across the width of the Noksan Station in the Nakdong River [15]. Bloom samples from this site were dominated by* M. aeruginosa* with MC-RR as the dominant variant. Spatial and temporal dynamics of phytoplankton communities was also studied in the Nakdong River. Small centric and pennate diatoms dominated from winter to early spring. A mixed community of cryptomonads, diatoms, and coenobial greens such as* Pediastrum* and* Scenedesmus* were dominant in late spring (May-June) while blue-green algae like* Anabaena*,* Microcystis,* and* Oscillatoria* dominated in summer (July–September). The role of hydrological changes was discussed as the main driving factor for phytoplankton succession as there was little fluctuation in the dominant phytoplankton even when the nutrient concentration varied in each study site [31]. It was concluded that the high loading of nutrients, the flow regulation by dams, and the estuarine barrage were responsible for bloom formations. In another study, data on the limnological parameters and phytoplankton population were collected over a long period of time (1993–2001) to investigate the relationship between dam hydrology and phytoplankton proliferations in the river [32]. Two phytoplankton species,* M. aeruginosa* and* Stephanodiscus hantzschii*, changed dynamically with dam hydrology during summer and winter, respectively, and the peaks of both species were observed when discharge persisted at low level. The authors argued that “smart flow control,” that is, more precise control of dam discharge during summer and winter, could prevent the bloom formation by the two species in the river systems and increase the efficiency of water resource management system.

Many dams have been constructed along the Han River for flood control, water supply, and hydropower generation. Fluctuations in the phytoplankton communities due to dam discharge have also been reported [33]. Recently, the effects of sampling periods and environmental factors on cyanobacterial communities at 6 sites along the Nakdong River were investigated in detail [34]. High-throughput sequencing of cyanobacterial 16S rRNA revealed a total of 175 cyanobacterial genera where cyanobacterial communities varied from June to September.* Prochlorococcus* was predominant in May, whereas the relative abundance of* Microcystis* and* Anabaena* increased with increase in water temperature. This shift in communities was mainly influenced by site location, nitrogen, and phosphorus.

## 3. Monitoring Approaches

An important goal of monitoring approach is the timely prediction of blooms events and therefore depends on various aspects such as flexibility, types of water bodies, dominant species, and sampling methods and time. Different sampling practices can influence the timely prediction of blooms and thus necessitate the development of a standardized procedure for sample collection. In a study by Ahn et al. [35], different sampling methods (pumping, integrating, Van Dorn, inflow, and mixing) and times were compared which revealed the integrating method as the most suitable one for sampling both* Microcystis* and* Oscillatoria*. In addition, the median and median absolute deviation (MAD) was proposed as a method to express a central tendency for cyanobacterial biomass.

Generally, microscopic identification and cell counting are the basic techniques for monitoring a cyanobacterial bloom. Various methods (boiling, vortexing, sonication, and TiO_2_ treatments) were compared for making single cell suspension of* Microcystis* colonies [36]. In this study, boiling was found to be the most suitable and effective method for generating free cells from colonies. Nutrients like N and P and their ratios were already reported to be reliable indicators of blooms in the Daechung Reservoir and are considered valuable in assessing the potential for future bloom development [37]. Later, K and Fe ratio was also proposed as a new parameter for predicting a bloom in the reservoir that was dominated by* Microcystis* sp. [38]. It was suggested that a threshold ratio (200) of K and Fe would reflect the same type of bloom as that with a cyanobacterial concentration of 20,000 cells/mL [39] and phycocyanin (PC) concentration of 20 pM [37].

**Figure 2:**
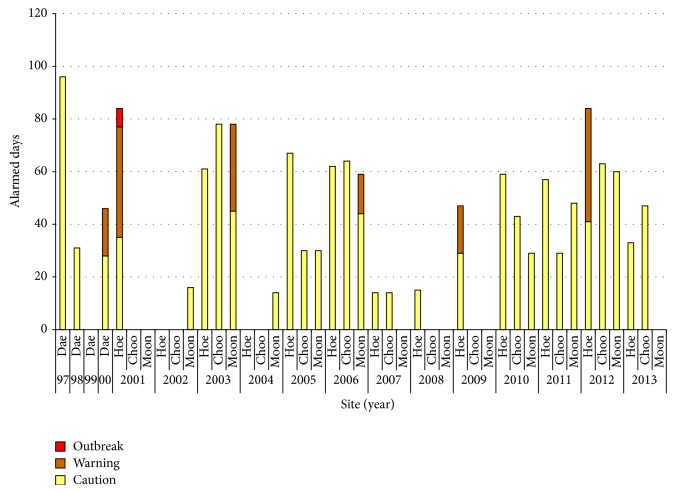
Alarmed days declared at 3 sites in Daechung Reservoir during 1997–2013, where Dae: Daechung Reservoir, Hoe: Hoenam, Choo: Choodong, and Moon: Moonue.

World Health Organization (WHO) has established a drinking water standard of 1 *μ*g/L for MC-LR and developed provisional guidelines as follows: Level 1 (low health risk probability): 20,000 cyanobacterial cells/mL or 10 *μ*g/L Chl* a* with dominance of cyanobacteria, Level 2 (moderate possibility of adverse health effects): 100,000 cells/mL or 50 *μ*g/L Chl* a*, and Level 3 (high health risk probability): formation of cyanobacterial scums [40, 41]. The Australian Drinking Water Guidelines, published jointly by the National Health and Medical Research Council and National Resource Management Ministerial Council, provide the frameworks for management of cyanobacteria and cyanotoxins in Australian water bodies [42]. Assuming the toxic cyanobacteria as the main bloom formers, an alert system for algal bloom was developed by the Ministry of Environment (South Korea) in 1997 (Table 2) [27] and since then has declared days of “caution/warning” at 3 sites in Daechung Reservoir during 1997–2013 (Figure 2) and in major Korean reservoirs (Table 3). The sequence of alert levels is based upon measurement of Chl* a* and cyanobacterial cell density (combined total of* Anabaena*,* Aphanizomenon*,* Microcystis,* and* Oscillatoria*). The alert is declared when Chl* a* and cyanobacterial cell density exceed the criteria consecutively two times. The analysis frequency is once per week which can be increased to over 2 times at “warning” and “outbreak.” Moreover, the alert is stepped down or cancelled if the Chl* a* level reaches below 15 *μ*g/L or cyanobacterial cell density is below 500 cells/mL. PC is a function of cyanobacterial biomass only, and its measurement seems to be a practical approach over that of Chl* a*. Therefore, an alternative cyanobacterial alert system based on PC level was suggested to monitor* Microcystis* bloom in Korean lakes [43]. This was based on PC levels of 0.1 (caution), 30 (warning), and 700 *μ*g/L (outbreak), respectively, and corresponded with the new suggested criteria of Chl* a* concentrations (3, 30 and 100 *μ*g/L) and cyanobacterial cell density (1,000, 10,000, and 100,000 cells/mL). In another study, a criterion of 10,000, 20,000, 40,000, and 80,000 cells/mL was also proposed for specific cyanobacteria like* Microcystis*,* Oscillatoria*,* Anabaena,* and* Aphanizomenon* spp., respectively. However, this was based on the cell numbers and cellular MCs content of cyanobacteria, which were collected from several Korean lakes and rivers [44].

## 4. Models for Prediction of Cyanobacterial Blooms

Community dynamics is often regulated by complex and diverse ecological parameters making it difficult to identify the underlying ecosystem mechanisms. Development of ecological models and computational technologies over the years has made the prediction of algal blooms more accurate. Various deductive and computational inductive models have been used for ecological modeling [45, 46]. Inductive models like artificial neuron networks (ANNs) have been widely used to forecast the occurrence of cyanobacterial blooms in reservoirs due to their better predictive power and its ability to map the nonlinear relationship between variables of the ecosystem [47]. ANN basically consists of interconnected processing elements having inputs that are multiplied by weights (strength of the respective signals) and an output layer. The weights of an artificial neuron can be adjusted using algorithms in order to obtain the desired output from the network. This process of adjusting the weights is called learning or training [48]. Backpropagation is the most common algorithm in which the signals are sent forward and the errors are propagated backwards. Multilayer perceptron (MLP) is a supervised learning algorithm having input, hidden layer(s) and an output layer. MLP with a backpropagation model has already been applied to predict the seasonal variations and the magnitude of bloom in South Korean water bodies. Environmental stressors and water quality indices can be checked with the use of an unsupervised learning algorithm like self-organizing maps (SOMs) which consists of an input and output layer.

Oh et al. [49] used these models for patterning algal communities and key factors causing bloom in Daechung Reservoir (located upstream of the Geum River) based on the 3-year data. These models were further used to explore the temporal shifts in environmental parameters and for predicting bloom peak in the reservoir [50]. Among the various environmental factors, water temperature and total dissolved nitrogen were found to be the major determinants for cyanobacteria and* Microcystis* bloom could be predicted 3 weeks earlier. The findings of this study also corresponded well with the alert system for prediction of cyanobacterial bloom in South Korea [27]. Although it is difficult to decide on the best training algorithm and the number of hidden layer nodes, its advantages lie in that it requires no information on the model structure and developed models were more flexible and adaptable to alternative scenarios [48].

Evolutionary algorithms (EAs) are another branch of machine learning techniques based on the principle of biological evolution, such as crossover, mutation, and chromosome's alteration, and are used to predict and elucidate specific ecological phenomena [51]. Various predictive models for algal blooms have also been developed and applied to the second largest river in South Korea (Nakdong River) [52, 53]. This is a eutrophic regulated river system with several multipurpose dams and is often faced with cyanobacterial blooms in the summer. A water quality model based on the USEPA's QUAL2E was also developed for management of large river systems [52]. Inclusion of some parameters like DO, BOD, nitrogen, and phosphorus in the model resulted in better agreement with the field measurements due to its ability to simulate the conversion of algal death to BOD, fixed plant DO, and the denitrification. Jeong et al. [53] modeled* M. aeruginosa* bloom dynamics using evolutionary computation with 25 limnological parameters. A nonlinear plankton model was developed that predicted daily abundance of phytoplankton species and the influence of environmental parameters was quantitatively analyzed [51]. They suggested that the relationship between river hydrology and phytoplankton dynamics should be explored over a longer period of time and water physicochemistry such as pH, temperature, and some nutrients played important roles in governing the daily changes of the two species (*M. aeruginosa* and* S. hantzschii*). Equation models based on a genetic programming (GP) algorithm and multiple linear regression (MLR) were used for predicting the temporal dynamics and magnitude of blooms. Although MLR failed to predict the bloom accurately, this study demonstrated that an inductive approach is more suitable for modeling the dynamics of algal blooms in a river-reservoir system. Later community changes of cyanobacteria were used to identify patterns in the eutrophication process of the river, revealing the seasonal occurrence of* Anabaena* bloom in spring and summer [10].

Two algorithms, an algebraic function model and a rule-based model, were developed with an aim to model the abundances of* M. aeruginosa* in Nakdong [54]. The rule-based model correctly predicted the timing and abundance of* Microcystis* on the basis of 8 years of limnological data from the lower Nakdong River. Sensitivity analysis basically provides useful information about the significant input variables and their relationship with each other and this study demonstrated high water temperature as the important parameter influencing the abundances of* M. aeruginosa*. Recently, SOM was used in river basin patterning and large sets of catchmentwise data (1655 stream sites) were explored in the Nakdong River to reveal stream modification patterns [55]. Stream Modification Index (SMI) system was developed for this purpose and the degree of stream modification was approximately related with sociogeographical aspects. The results of this survey provided an insight into the morphological characterization and status of streams or rivers in the river basin.

More recently, Cha et al. [11] developed a Bayesian hurdle Poisson model based on the data from 2007 to 2011 to predict cyanobacterial bloom in Lake Paldang. The model predictions demonstrated that the principal factor that determines the success of cyanobacteria was temperature. High temperature and a stable water column were demonstrated as main factors required for high abundance of cyanobacteria. This model can be used to forecast cyanobacteria and for the development of mitigation strategies of cyanobacterial blooms.

## 5. Conclusion

Reservoirs and regulated rivers are the major sources of freshwater in South Korea. Various field studies have been done and technical approaches have been adopted to solve the nationwide problem of cyanobacterial blooms. The phytoplankton community shows seasonal variation in most of the reservoirs and has been related to temperature, nutrients like P, and changes in hydrology.* Microcystis*,* Anabaena,* and* Oscillatoria* have been predominantly documented from most South Korean reservoirs. However, cyanobacterial growth and dispersal could be studied in relation to land use and climate change. Furthermore, monitoring techniques and alert systems have also been developed for the timely prediction and management of cyanobacterial blooms. However, inclusion of other parameters like PC as an alert criterion can prove useful for efficient monitoring. The levels of cyanotoxins especially MCs are reported to be below the WHO limit of 1 *μ*g/L in most of the reservoirs and till now there is no data on human health hazards due to exposure to toxic cyanobacterial blooms in South Korea. However, a standard for cyanobacterial biomass and selected cyanotoxins would be important in the context of development of management strategies and water safety plan.

## Figures and Tables

**Table 1:** Toxins concentrations reported from various freshwater bodies in South Korea.

Dominant cyanobacteria genus	Toxins (*μ*g/g or *μ*g/L)	Reservoir (river)	Sampling date	Reference
*Microcystis *	133 (*μ*g/g, MC)	Soyang (Han River)	26 Oct. 1992	[13]
*Oscillatoria *	76	Daechung (Geum River)	06 Oct. 1992	
*Anabaena *	115	Jangsong (Yeongsan River)	15 Oct. 1994	
(algal samples)	635	Noksan (Nakdong River)	27 Aug. 1995	

*Microcystis* (algal samples)	288–2612 (*μ*g/g, MC)	10 large reservoirs	Aug. 1996–Oct. 1997	[14]

*Microcystis *	1.89 *μ*g/L except at Site 1 (19.1 *μ*g/L)	Noksan Station (Nakdong River)	20 Aug. 1998	[15]

*Microcystis *	0.057–0.488 (*μ*g/L, MC)	Paldang (Han River)	03 Sep.–28 Nov. 1997	[8]

*Microcystis *	0.2 (*μ*g/L, MC)	Daechung (Geum River)	27 Apr.–12 Oct. 1999	[16]

*Anabaena* *Oscillatoria *	0.01–0.08 (*μ*g/L, AT)	Daechung (Geum River)	18 Jun.–5 Nov. 2001	[17]

*Microcystis *	0.59 (*μ*g/L, MC) 0.55 (*μ*g/L, MC)	Yangsoori Seokchon (Han River)	2-3 Oct. 2004	[18]

MC: microcystin, AT: anatoxin-a.

**Table 2:** Alert levels framework for algal bloom in South Korea.

Level	Caution	Warning	Outbreak
Chlorophyll-*a* (*μ*g/L)	≥15	≥25	≥100
Cyanobacteria (cells/mL)	≥500	≥5,000	≥1,000,000
Monitoring interval (/week)	1	2	2

**Table 3:** Days of “caution (warning)” declared in major Korean reservoirs (Ministry of Environment, [19]).

Reservoir	Year										
	'02	'03	'04	'05	'06	'07	'08	'09	'10	'11	'12
**Sum**	**93**	**166 (33)**	**97**	**318**	**311 (34)**	**198 (97)**	**114 (19)**	**244 (43)**	**177 (14)**	**131**	**161 (43)**
Han River	—	—	—	—	31	—	11	—	—	—	14
Paldang	20	—	14	15	21	—	36	23	43	—	28
Gwanggyo	—	—	—	—	—	12	—	—	—	—	35
Unmun	—	—	—	—	—	—	—	—	—	16	—
Daechung	16	90 (33)	14	67	78 (15)	14	15	47 (18)	59	57	84 (43)
Yongdam	—	—	—	80	38	—	—	—	19	—	—
Boryeong	—	—	—	—	—	—	—	—	19 (14)	—	—
Yeongcheon	—	—	40	86	100 (19)	139 (97)	37 (19)	—	—	36	—
Juam	57	76	14	—	—	19	—	41 (25)	37	—	—
Dongbok	—	—	15	—	—	14	—	41	—	—	—
Jinyang	—	—	—	17	—	—	15	51	—	—	—
Deokdong	—	—	—	—	—	—	—	—	—	22	—
Angye	—	—	—	53	43	—	—	—	—	—	—
Hoeya	—	—	—	—	—	—	—	41	—	—	—
